# Supplementary material for: Features of Profiles of Biologically Active Compounds of Primary and Secondary Metabolism of Lines from VIR Flax Genetic Collection, Contrasting in Size and Color of Seeds
Source: Plants (Basel). 2022 Mar 11;11(6):750. doi: 10.3390/plants11060750 (PMC8953942; doi:10.3390/plants11060750)
Supplement: Supplementary file 1 [file plants-11-00750-s001.zip › TableS3.pdf]

Table S3. Comparison of the fatty acid composition in seeds and oil of the VIR genetic collection lines, % of total fatty acids amount.

Lines of genetic collection	Seedflour					Seed oil (Porokhovinova et al., 2017b [47])				
	**pal**	**ste**	**ole**	**lio**	**lin**	**pal**	**ste**	**ole**	**lio**	**lin**
gc -2	9,4	3,9	33,8	23,5	29,4	4,3	3,1	20,4	14,4	57,8
gc -65	8,4	5,0	36,0	26,3	24,3	4,3	3,9	22,3	19,8	49,6
gc -109	7,9	3,6	36,8	23,1	28,7	4,9	3,5	29,8	15,6	46,2
gc -119	9,2	4,5	31,6	23,5	31,3	5,9	4,0	39,1	18,1	32,9
gc -124	6,5	4,1	35,4	22,7	31,4	4,2	3,0	22,2	19,2	51,4
gc -129	8,1	3,5	29,6	24,6	34,2	6,2	3,8	19,5	16,4	54,0
gc -136	8,2	4,3	28,6	21,6	37,3	5,2	4,8	14,9	14,3	60,8
gc -141	7,7	3,6	46,5	26,7	15,5	5,4	4,3	25,4	15,5	49,4
gc -159	11,6	6,3	19,0	25,0	38,1	4,0	3,3	23,0	17,4	52,2
gc 173	9,4	5,8	27,8	25,5	31,5	5,1	3,7	17,4	18,7	55,1
gc-391	10,0	4,4	6,1	64,8	14,8	6,4	4,9	27,4	56,6	4,6
average										
Correlations<br>n=11<br>n=10 (witoutgc-391)						-0,03<br>-0,24	-0,13<br>-0,15	0,02<br>0,24	0,99<br>0,37	0,62<br>0,24
