# Supplementary material for: Features of Profiles of Biologically Active Compounds of Primary and Secondary Metabolism of Lines from VIR Flax Genetic Collection, Contrasting in Size and Color of Seeds
Source: Plants (Basel). 2022 Mar 11;11(6):750. doi: 10.3390/plants11060750 (PMC8953942; doi:10.3390/plants11060750)
Supplement: Supplementary file 1 [file plants-11-00750-s001.zip › TableS4.pdf]

Table S4. Amino acid composition of flax seed proteins according to the results of metabolomic analysis and standard methods [24, 29]. * - not detected in the metabolic analysis

	Oomah93 [24], g/100g protein						Bhatty90 [29], g/16gN			Metabolome mg/g seeds	
Amino acid	Nor-Lin	Fos-ter	Ome-ga	Average	%	**% witout "*"**	Volume	%	**% witout "*"**	Volume	**% witout "*"**
Aspartic acid	9.3	10	9,7	9,7	11	**13**	12,5	10	**14**	21,0	**18**
Threonine	3.6	3,8	3,7	3,7	4	**5**	4,9	4	**5**	6,0	**5**
Serine	4.5	4,7	4,6	4,6	5	**6**	5,8	5	**6**	2,7	**2**
Glutamic acid	19.6	20	19,7	19,8	22	**28**	26,3	22	**29**	19,6	**16**
Proline	3.5	3,8	3,5	3,6	4	**5**	5,2	4	**6**	20,0	**17**
Glycine	5.8	5,9	5,8	5,8	6	**8**	7,0	6	**8**	10,4	**9**
Alanine	4.4	4,7	4,5	4,5	5	**6**	5,4	5	**6**	14,2	**12**
Cystine*	1.1	1,8	1,1	1,3	1		3,8	3			
Valine	4.6	5,1	4,7	4,8	5	**7**	5,6	5	**6**	6,9	**6**
Methionine*	1.5	1,4	1,4	1,4	2		2,2	2			
Isoleucine*	4	4,1	4	4,0	4		5,2	4			
Leucine	5.8	6	5,9	5,9	6	**8**	6,8	6	**8**	4,7	**4**
Tyrosine	2.3	2,4	2,3	2,3	3	**3**	2,9	2	**3**	2,5	**2**
Phenylalanine	4.6	4,8	4,7	4,7	5	**7**	5,3	4	**6**	9,8	**8**
Histidine	2.2	2,1	2,3	2,2	2	**3**	2,9	2	**3**	1,1	**1**
Lysine*	4	4	3,9	4,0	4		4,1	3			
Arginine*	9.2	10	9,4	9,5	10		11,8	10			
Tryptophan*							1,8	2			
Summ	90.0	94,6	91,2	91,9	100	**100**	119,5		**100**	119,0	**100**
correlation with Oomah93						**1,00**			**1,00**		**0,62**
correlation with Bhatty90						**1,00**			**1,00**		**0,60**

* - not detected in the metabolic analysis
